# NGF administration is associated with increased GnRH immunoreactivity and a GnRH-associated phenotype in hypothalamic NSCs of aging mice

**DOI:** 10.3389/fendo.2026.1736356

**Published:** 2026-04-24

**Authors:** Jiao Luo, Junhai Jiang, Tiantian Zhang, Xiaohua Zhang, Yadong Huang, Xintao Zhang, Yulong Wang, Yan Yang

**Affiliations:** 1 Department of Rehabilitation Medicine, Dapeng New District Nan’ao People’s Hospital, Rehabilitation Branch of the First Affiliated Hospital of Shenzhen University, Shenzhen, China; 2 Department of Rehabilitation, the First Affiliated Hospital of Shenzhen University, Shenzhen Second People’s Hospital, Shenzhen, China; 3 Guangdong Reading Biotech Co., Ltd, Guangzhou, China; 4 Department of Cell Biology, Jinan University, Guangzhou, China; 5 Guangdong Province Key Laboratory of Bioengineering Medicine, Guangzhou, China; 6 Department of Urology, The First Affiliated Hospital of Shenzhen University, Shenzhen Second People’s Hospital, Shenzhen, China

**Keywords:** aging, differentiation, GnRH, hypothalamic neural stem cells, nerve growth factor

## Abstract

**Purpose:**

Age-related decline in testosterone is closely associated with hypothalamic gonadotropin-releasing hormone (GnRH) neuron dysfunction. Nerve growth factor (NGF) has emerging roles in reproductive system. However, its effect on the differentiation of hypothalamic neural stem cells (htNSCs) into GnRH-associated phenotype remains unexplored.

**Methods:**

Male senescence-accelerated mouse P8 (SAMP8) models of age-related hypogonadism received a single intracerebroventricular injection of NGF at 6.25, 12.5, and 25 μg/kg. Serum levels of luteinizing hormone (LH), follicle-stimulating hormone (FSH), and testosterone were measured 8 h, 24 h, 96 h, 7 d and 10 d post-injection. A kisspeptin agonist and antagonist were used in combination to interrogate whether NGF’s endocrine effects depend on canonical kisspeptin signaling. *In vivo* GnRH immunoreactivity and Sox2/GnRH co-localization in the organum vasculosum of the lamina terminalis (OVLT) were evaluated by double immunofluorescence as phenotypic readouts. In parallel, a three-dimensional culture system was employed to evaluate NGF-induced differentiation of primary htNSCs into GnRH-secreting neurons. Transcriptomic profiling and quantitative PCR were used to identify key signaling pathways involved.

**Results:**

Central NGF administration significantly elevated serum FSH, LH, and testosterone levels in male SAMP8 mice, and these effects were not evidently mediated by canonical kisspeptin signaling. In the OVLT, NGF was associated with increased GnRH immunoreactivity and Sox2/GnRH co-localization, which may reflect phenotypic remodeling toward a GnRH-related cellular state. *In vitro*, NGF promoted htNSCs differentiation toward a GnRH-associated neuroendocrine phenotype, accompanied by increased *Gnrh1* expression/GnRH release and enrichment of neuropeptide-related signaling pathways.

**Conclusion:**

These findings suggest that central NGF administration may be associated with an increase in GnRH-immunoreactive cellular phenotypes, accompanied by enhanced the hypothalamic-pituitary-testicular axis endocrine output in male SAMP8 mice. Definitive *in vivo* neurogenesis will require future validation using proliferation and lineage-tracing approaches.

## 1 Introduction

Testosterone levels in men decline with advancing age, contributing to reduced reproductive function. The global aging population has increased the demand for effective strategies to elevate sex hormone levels. Clinically, exogenous testosterone supplementation (1) is often used to restore androgen levels, improving sexual activity, erectile function, and certain aspects of physical performance. However, prolonged administration of testosterone via implants carries significant risks of infertility (2).

The biosynthesis of testosterone is intricately regulated by the hypothalamic-pituitary-testicular (HPT) axis, with gonadotrophin-releasing hormone (GnRH) as the master regulator (3). Mature GnRH neurons in the hypothalamus secrete GnRH in a pulsatile manner, stimulating the pituitary gland to produce luteinizing hormone (LH) and follicle stimulating hormone (FSH), which are essential for testosterone production and sperm maturation. Functional studies have demonstrated that although pituitary responsiveness to GnRH remains largely intact during aging, a significant decline in pulse amplitude and promoter activity of the *Gnrh1* gene, the amplitude and circadian rhythmicity of LH and testosterone secretion are both blunted (4). Importantly, aging activates hypothalamic nuclear factor kappaB signaling, which inhibits GnRH transcription and contributes to reduced GnRH output. Restoration of GnRH reversed aging-associated neurogenic deficits, suggesting that hypothalamic GnRH decline is both a hallmark and potential driver of aging phenotypes (5). Additional studies in rodents and primates show that aging is accompanied by reduced GnRH mRNA expression, diminished neuronal activation in response to stimulatory cues, and blunted hormonal surges (5, 6). Together, these findings indicate that aging leads to both quantitative and qualitative impairments in hypothalamic GnRH function. Thus, strategies that restore or reinforce rhythmic GnRH signaling may offer a promising alternative for enhancing reproductive function during aging.

Neural stem/progenitor cells (NSCs) in the adult brain possess the potential to differentiate into functional neurons, providing a promising therapeutic approach. Previous studies (7–9) had developed protocols to generate neuropeptidergic hypothalamic neurons from human stem cells and induced pluripotent stem cells. Various stimuli, including anti-Müllerian hormone (AMH), growth hormone (GH), and insulin-like growth factor 1 (IGF1), have been implicated in the development of GnRH neurons (10) and the differentiation of NSCs. Promoting hypothalamic NSCs (htNSCs) toward a GnRH-associated neuroendocrine phenotype using biological factors may represent a strategy to compensate for the age-related decline in GnRH function. Nevertheless, the regulatory effects and molecular mechanisms governing htNSC differentiation toward GnRH phenotypes remain incompletely understood and warrant further investigation.

Nerve growth factor (NGF), a well-characterized neurotrophic factor, plays a crucial role in neuronal development, survival, and proliferation. Studies has demonstrated significant therapeutic potential of NGF in age-related neurodegenerative diseases such as Alzheimer’s disease (AD) (11) through TrkA/p75NTR receptors. While numerous studies have elucidated NGF’s role in regulating the differentiation of NSCs (12, 13), its specific involvement in directing htNSCs toward reproductive hormone secretion has not been fully explored.

In this study, we investigated whether central NGF administration influences htNSC differentiation and enhances HPT axis activity in 10-month-old male SAMP8 mice, a model exhibiting age-related hypogonadotropic hypogonadism. Our findings provide preclinical evidence supporting NGF as a potential strategy for improving GnRH-associated neuroendocrine function and ameliorating GnRH-related hypogonadism during aging.

## 2 Materials and methods

### 2.1 Animals

Adult BALB/c mice (8 weeks old) were purchased from Beijing HFK Bioscience Co., Ltd. Male SAMP8 mice (8 months old) were purchased from the Third Affiliated Hospital of Tianjin University of Traditional Chinese Medicine. All animals were housed under specific pathogen-free conditions at 24 °C ± 2 °C with a 12-hour light/dark cycle and 50–60% humidity. The SAMP8 mice were housed individually during experimentation to prevent aggressive behavior and were randomly assigned to experimental groups using a random number generator in Excel. In this study, animal euthanasia was euthanized via intraperitoneal injection of pentobarbital sodium (Sinopharm Chemical Reagent Co., Ltd., Shanghai, China) at a dose of 100 mg/kg. The euthanasia process was performed in strict accordance with the ethical guidelines established by the Institutional Animal Care and Use Committee of Shenzhen University. The work has been reported in line with the ARRIVE guidelines 2.0.

### 2.2 Intracerebroventricular injection of NGF

**Figure 1:**
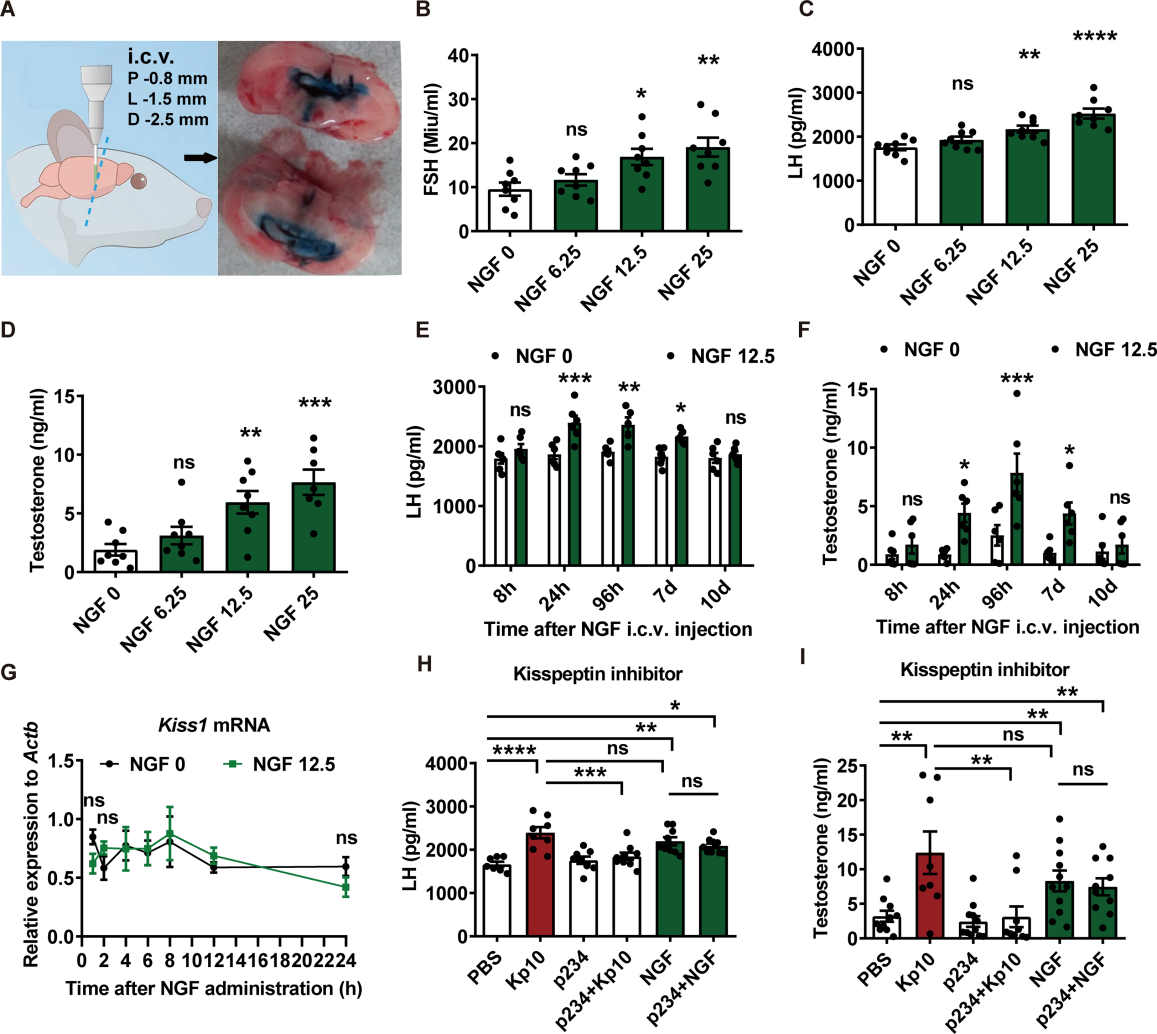
A single i.c.v. injection of NGF increased sex hormone levels in aging male SAMP8 mice. **(A)** Schematic diagram showing the verification of i.c.v. injection using 0.4% trypan blue into the lateral cerebral ventricle at the following coordinates: -0.8 mm posterior to bregma, -1.5 mm lateral to midline and -2.5 mm deep to cranial surface. **(B–D)** Serum levels of FSH, LH, and testosterone in 10-month-old male SAMP8 mice at 7 days following a single i.c.v. injection of NGF at 6.25, 12.5, and 25 μg/kg (n = 8). One-way ANOVA. **(E, F)** Time-course changes in serum LH and testosterone levels following a single i.c.v. injection of NGF (12.5 μg/kg) (n = 6). Two-way ANOVA. **(G)** Relative expression of hypothalamic *Kiss1* mRNA after NGF injection (12.5 μg/kg) (n = 6). Two-way ANOVA. **(H, I)** Serum LH and testosterone levels at 24 h post-treatment with NGF (50 μg/kg, i.c.v.), kisspeptin agonist Kp10 (40 ng/kg, i.c.v), or vehicle, with or without pretreatment with the kisspeptin antagonist p234 penetratin (p234-p, tail vein injection) (n = 10). One-way ANOVA. Data are presented as mean ± SEM. ****P< 0.0001, ***P< 0.001, **P< 0.01, *P< 0.05 compared to SAMP8 mice with vehicle (NGF 0 μg/kg); “ns” indicates no significant difference.

10-month-old male SAMP8 mice, previously reported to exhibit significantly lower androgen levels compared to age-matched control strains, were used as a model for age-related hypogonadotropic hypogonadism (14). During the i.c.v. injection surgery, mice were anesthetized with 2% isoflurane (cat. no. R510-22-10, RWD Life Science, China) for induction and maintained on 1% isoflurane using a Small Animal Anesthesia Machine (Model R500, RWD Life Science, China). Recombinant mouse beta-NGF protein (cat. no. 1156-NG/CF, R&D system, Abingdon, UK), carrier free, was initially reconstituted at a concentration of 250 μg/ml in sterile 0.01 M phosphate-buffered saline (PBS), and subsequently diluted to the desired working concentrations for i.c.v. injection. For the control group, mice received an equivalent volume of sterile 0.01 M PBS, the same solvent used to dissolve NGF. A single i.c.v. injection of NGF (6.25, 12.5, or 25 μg/kg) was administered into the lateral cerebral ventricle (coordinates: -0.8 mm posterior to bregma, -1.5 mm lateral to midline and -2.5 mm deep to cranial surface). Trypan blue (0.4%) was used only in separate preliminary verification injections as a visual tracer to confirm stereotaxic coordinates and successful ventricular delivery (**Figure 1A**). For experimental infusions, NGF was prepared at the desired concentration and delivered in a final volume of 3 μl at a rate of 1 μl/min using a stereotaxic apparatus (cat. no. 68802, RWD Life Science).

### 2.3 Kisspeptin agonist and antagonist administration

NGF was administered via i.c.v. injection at a dose of 25 μg/kg. The kisspeptin agonist, Kisspeptin-10 (Kp10) (cat. no. 048-56, Phoenix Pharmaceuticals Inc., Belmont, CA, USA), was dissolved in PBS and injected into the lateral cerebral ventricle at a dose of 40 ng/kg using the same i.c.v. procedure as NGF. The kisspeptin antagonist p234 penetratin (hereafter referred to as p234-p; cat. no. 048-96, Phoenix Pharmaceuticals Inc., Belmont, CA, USA), was dissolved in PBS and administered by intravenous injection via the tail vein injection (40 μg/kg), 1 hour prior to i.c.v. injection of NGF or Kp10. Serum samples were collected from treated mice 24 hours after the final injection for hormone analysis. Serum samples were collected from treated mice 24 hours between 17:00 and 19:00 after the final injection for hormone analysis.

### 2.4 Hormone concentrations of serum

All blood samples from SAMP8 mice were collected via retrobulbar venous plexus between 17:00 and 19:00 (early-to-mid scotophase). The serum was separated by centrifuge at 3000 rpm for 15 minutes. Testosterone levels in serum were measured using I^125^-testosterone Coat-A-Count RIA kits (cat. no. S10940093, Beijing North Institute of Biological Technology) (15). Serum levels of LH and FSH were quantified using a sensitive sandwich ELISA kit (cat. no. CSB-E12770m, CUSABIO, Wuhan) or competitive ELISA kit (cat. no. CSB-E06871m, CUSABIO, Wuhan) (16), following the manufacturer’s instructions, respectively.

### 2.5 Primary htNSC isolation, culture, and three-dimensional differentiation

Primary culture of htNSCs was performed as previously described (17). In brief, the hypothalamus was dissected from newborn male BALB/c mice within 3 days, cut into small pieces (∼1 mm^3^), and digested with TrypLE™ Express enzyme (cat. no. 12605-010, Life Technologies) for 10 minutes. The digestion was halted using PBS, followed by centrifugation at 1000 rpm, 3 minutes. Cells were suspended in htNSCs medium containing Neurobasal-A (cat. no. 10888-022, Invitrogen), 2% B27 without vitamin A (cat. no. 12587010, Invitrogen), 0.25% GlutaMAX™ supplement (cat. no. 35050061, Invitrogen), 10 ng ml^−1^ EGF (cat. no. PMG8041, Invitrogen), 10 ng ml^−1^ bFGF (cat. no. 10014-HNAE, Sino Biological), and 1% penicillin-streptomycin (cat. no. 15240062, Invitrogen). The cells were seeded in ultralow-adhesion 6-well plates (cat. no. 3471, Corning) and incubated in 5% CO_2_ at 37◦C. After 3 days, neurospheres were collected by centrifugation and dissociated into single cells using TrypLE Express enzyme. The cells were proliferated and maintained in htNSCs medium for three passages prior to the differentiation experiments.

For the 3D differentiation of htNSCs, thin-layer 3D cultures were conducted as previously described (18, 19). 3D-htNSCs differentiation were performed according to a previous study (7). To prepare the Matrigel working solution for plate pre-coating, Matrigel was thawed on ice and diluted 1:100 in cold Neurobasal-A medium. The working solution was added to fully cover the surface of the culture plates and incubated in a 37°C CO_2_ incubator for at least 1 hour. For 3D culture, a single-cell suspension of htNSCs was mixed with Matrigel (Cat. No. 356231, BD Biosciences) at a 1:1 ratio and promptly seeded onto the pre-coated plates (100 μl containing 1 × 10^7^ cells/well for 24-well plates; 400 μl containing 4 × 10^7^ cells/well for 6-well plates) to form a thin-layer 3D matrix (approximately 100-300 μm) at a relatively high cell density. After overnight incubation, the htNSC medium was refreshed with differentiation medium I composed of Neurobasal-A with 2% B27™ (cat. no. 17504044, Invitrogen), 0.5% GlutaMAXTM supplement, 1% penicillin-streptomycin, 1% N-2 Supplement (cat. no. 17502001, Invitrogen), and 200 ng ml^−1^ DAPT (cat. no. HY-13027, MedChemExpress), and cultured for 4 days. From days 4 onward, differentiation medium I was replaced with differentiation medium II, composed of Neurobasal-A with 2% B27™, 0.5% GlutaMAXTM supplement, 1% penicillin-streptomycin, 1% N-2 Supplement, and 20 ng ml^−1^ BDNF (cat. no. 50240-MNAS, Sino Biological). The medium was changed by replacing half of its volume every three days until next analysis. Different concentrations of recombinant mouse NGF (cat. no. 1156-NG/CF, R&D system, Abingdon, UK) were added to the differentiation medium to induce htNSC differentiation (7). Additionally, GnRH concentrations in the culture supernatant of differentiating htNSCs were measured with a mouse GnRH ELISA Kit (cat. no. RD-GnRH-Mu; Reddot Biotech Inc., Canada) according to the manufacturer’s protocol.

### 2.6 Immunofluorescence staining

A primary single-cell suspension of htNSCs was seeded onto sterile glass coverslips placed in 24-well plates. After 24 hours of culture, the cells were fixed with 4% paraformaldehyde (PFA) for 15 minutes at room temperature and subjected to immunofluorescence staining to detect stem neural markers (Sox2 and Nestin) and NGF receptors (TrkA and p75NTR). For 3D-htNSCs differentiation assays, cells were fixed with 4% PFA for 60 minutes between days 8 and 21 days of induction and stained for neural markers and neuropeptides. Fixed htNSCs were blocked using QuickBlock™ buffer for (cat. no. P0260, Beyotime) at 37 °C for 30 minutes, followed by overnight incubation at 4 °C with primary antibodies. After three washes with PBS, the samples were incubated with appropriate fluorescent secondary antibodies for 60 minutes at room temperature. Details of the primary and secondary antibodies are provided in **Table 1**. Finally, sample were mounted with antifade medium containing DAPI (cat. no. P0131, Beyotime) to preserve fluorescence and counterstain nucleus. Immunofluorescence staining of htNSCs was visualized using a Confocal Laser Scanning Microscope (Olympus FV3000, Japan) equipped with a 20× objective. Images were acquired using single-plane model. For quantitative analysis, five immunofluorescence-stained images were randomly selected per sample from double-stained fields containing Tuj1^+^/GnRH^+^ or Map2^+^/GnRH^+^ cells. The number of GnRH-positive and Tuj1- or Map2-positive neurons was manually counted, and results were expressed as a percentage of the total number of DAPI-stained nuclei in each field.

**Table 1:** Primary and secondary antibodies used for immunofluorescence.

Primary antibodies	Manufacturer	Catalog number; dilution
Rabbit polyclonal anti-GnRH	Affinity	DF8553; 1:200
Mouse monoclonal anti-Sox2	R&D Systerms	MAB 2018; 1:100
Anti-Nestin antibody [SP103]	Abcam	ab105389; 1:200
Rabbit monoclonal anti-TrkA	Abcam	ab109010; 1:200
Rabbit monoclonal anti-p75NTR	Abcam	ab52987; 1:50
Mouse monoclonal anti-Tuj1	Cell Signaling Techology	4466S; 1:200
Chicken polyclonal anti-Map2	Biolegend	822501; 1:5000
Goat anti-Rabbit IgG (H+L) Highly Cross-Adsorbed Secondary Antibody, Alexa Fluor Plus 555	Thermo Fisher Scientific	A32732; 1:1000
Goat anti-Mouse IgG (H+L) Cross-Adsorbed Secondary Antibody, Alexa Fluor 488	Thermo Fisher Scientific	A-11001; 1:1000
Goat anti-Rabbit IgG (H+L) Cross-Adsorbed Secondary Antibody, Alexa Fluor 488	Thermo Fisher Scientific	A-11008; 1:1000
Goat anti-Mouse IgG (H+L) Cross-Adsorbed Secondary Antibody, Alexa Fluor™ 555	Thermo Fisher Scientific	A-21422; 1:1000

For *in vivo* analysis, SAMP8 mice were anesthetized with intraperitoneal injection of pentobarbital sodium at a dose of 80 mg/kg and perfused transcardially with 10 ml of cold PBS, followed by 20 ml of 4% PFA over 5 minutes. Brains were carefully dissected and post-fixed in 4% PFA overnight at 4 °C. The tissue was then sequentially dehydration in 20% and subsequently 30% sucrose solutions until fully equilibrated. Brains tissues were embedded in OCT compound and stored at −20 °C. Specifically, coronal cryosections were prepared using a cryostat (Thermo Scientific HM525 NX, USA). As described in previous studies (20, 21), coronal serial sections spanning from the medial septum through the preoptic area to the median eminence, encompassing the hypothalamic region, were cut at 35 μm in SAMP8 mice for the quantification of GnRH neurons using a rabbit polyclonal anti-GnRH antibody, and at 16 μm thickness for double immunofluorescence analysis of GnRH and Sox2. Fluorescence signals were visualized using a fluorescence microscope (Olympus IX70, Japan) and a Confocal Laser Scanning Microscope (Leica TCS SP8, German), both equipped with a 20× objective. Single-plane images were acquired to visualize GnRH-positive cell bodies. For each animal, GnRH-positive neurons were quantified in six consecutive 35-μm-thick coronal sections encompassing the OVLT region.

### 2.7 RNA extraction and real-time PCR analysis

Total RNA was extracted using TRIzol reagent (cat. no. 15596026, Invitrogen) from NGF-treated or vehicle-treated htNSCs, as well as from the whole hypothalamic tissue isolated from SAMP8 mice. A total of 1 µg of RNA from 3D-htNSCs was reverse transcribed into cDNA using the RevertAid RT Reverse Transcription Kit (cat. no. K1691, Thermo Scientific™). Quantitative PCR (qPCR) analysis was performed using SYBR Green Mix (Cat.QPK-201, Toyobo). Data were collected with the Bio-Rad CFX Connect Real-Time system and normalized against *Actb* in triplicate. Primers were synthesized by Suzhou Hongxun Biotechnology (**Supplementary Table 1**).

### 2.8 RNA-sequencing and bioinformatics analysis

RNA samples were transported on dry ice and sequenced at Novogene Bioinformatics Technology Co., Ltd. Briefly, RNA integrity was assessed using the Agilent 2100 Bionalyzer. Sequencing libraries were prepared using the NEBNext^®^ Ultra™ RNA Library Prep Kit for Illumia^®^, following the manufacturers’ protocol. Sequencing was performed on an Illumina Novaseq platform, generating 150 bp paired-end reads. Clean data were generated from the raw sequencing data after quality control (**Supplementary Table 2**). Mapping analysis was performed using HISAT2 (V2.05), quantification using FeatureCounts (1.5.0-p3), differential expression analysis using DESeq2 (V1.20.0), and Gene Ontology (GO) and Kyoto Encyclopedia of Genes and Genomes (KEGG) enrichment analysis using clusterProfiler (V3.8.1). Differentially expressed genes (DEGs) were identified base on |log2FoldChange| ≧4.0 and adjusted *p*-value (padj) ≦0.01. Significance of enriched GO and KEGG terms was set at *p*-value≦0.05. Our RNA-sequencing data have been deposited in the Genome Sequence Archive under the accession number CRA019629.

### 2.9 Statistical analysis

All data were analyzed using GraphPad Prism 8.0 and are expressed as mean ± SEM. Data were compared by an unpaired Student’s *t*-test between two groups, one-way ANOVA analysis of variance for multiple comparisons of different dose groups, and a two-way ANOVA analysis of variance for the experimental design involves two independent factors: treatment (NGF vs. Vehicle) and time. A *p*-value <0.05 was considered statistically significant in all cases. “*” represents *P* < 0.05, “**” represents *P* < 0.01, “***” represents *P* < 0.001, “****” for *P* < 0.0001, and “ns” for no significant difference. The number of biologically independent experiments, as well as the ages and sexes of animals, are provided in the figure legends.

## 3 Results

### 3.1 A single i.c.v. injection of NGF elevated androgen levels in aging male SAMP8 mice

A single i.c.v. injection of NGF (6.25, 12.5, or 25 μg/kg) induced a dose-dependent increase in serum levels of FSH, LH, and testosterone in 10-month-old male SAMP8 mice (**Figures 1B–D**). Temporal analysis following 12.5 μg/kg NGF injection revealed significant elevations in LH and testosterone at 24 hours, 96 hours, and 7 days post-injection (**Figures 1E, F**). Although NGF has been reported to exhibit a relatively short half-life in the brain (approximately 6–8 hours), its endocrine effects persisted for up to 7 days, suggesting sustained activation of upstream neuroendocrine output and supporting a prolonged modulatory influence of NGF on HPT-axis hormone homeostasis.

Given that kisspeptin is a critical upstream regulator of hypothalamic GnRH secretion, we next examined whether NGF-induced endocrine changes were mediated through kisspeptin signaling. NGF treatment did not alter *Kiss1* mRNA expression within 24 hours (**Figure 1G**), arguing against a prominent transcriptional activation of kisspeptin at this time points. To further assess the functional relationship, mice were treated with NGF in the presence or absence of the kisspeptin agonist Kp10 and the antagonist p234-p. Both NGF and Kp10 significantly increased serum LH and testosterone levels when administered alone. Notably, the kisspeptin antagonist p234-p effectively blocked the hormonal response induced by Kp10 but failed to attenuate the NGF-induced elevation in hormone levels (**Figures 1H, I**). These findings indicate that NGF-driven endocrine activation in this model is not primarily mediated by canonical Kisspeptin/Kiss1r signaling under the tested conditions.

### 3.2 NGF administration increased GnRH-immunoreactive cells and Sox2/GnRH co-expression in the OVLT of aging SAMP8 mice

**Figure 2:**
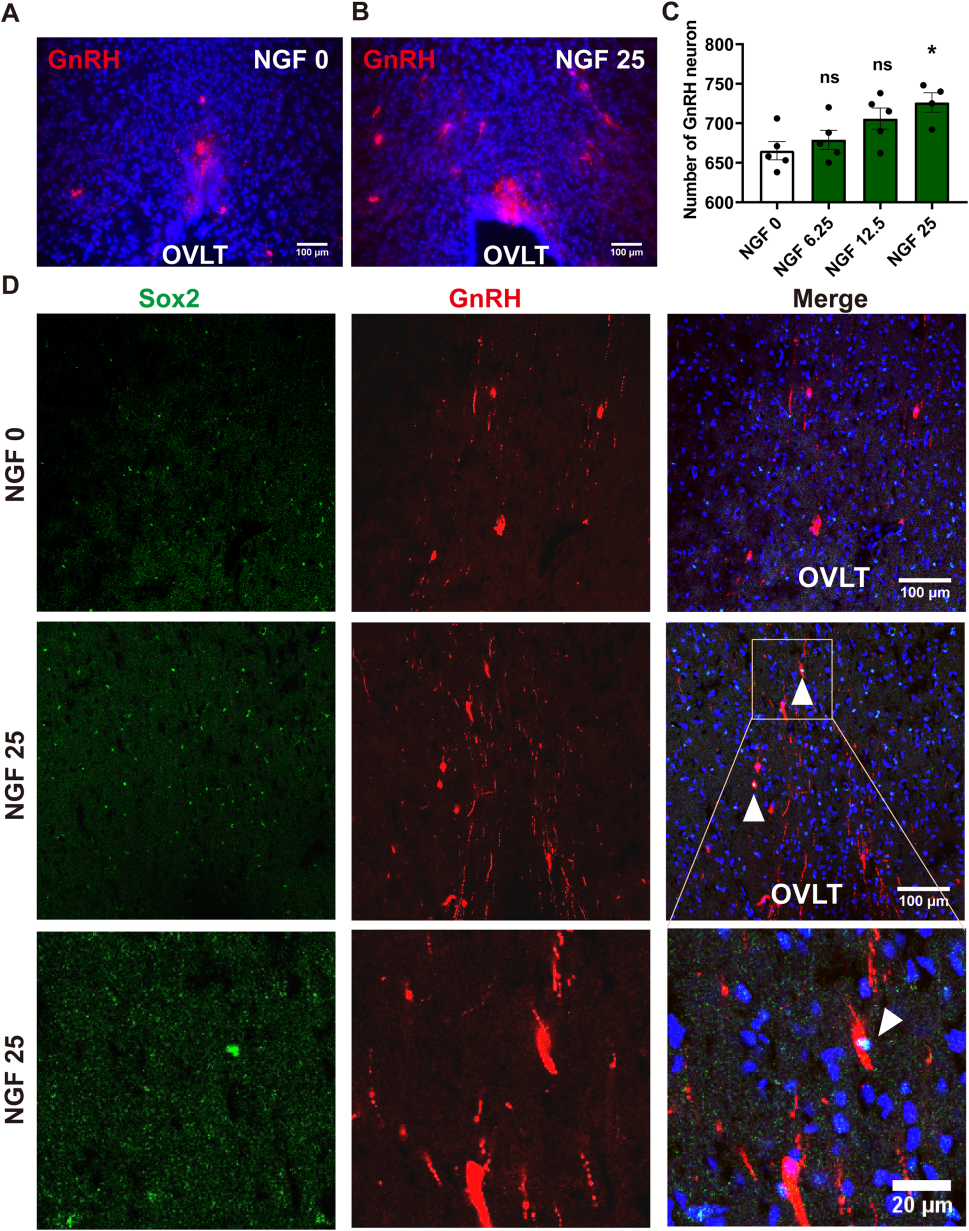
NGF increased GnRH-immunoreactive (GnRH^+^) cells and induced Sox2/GnRH co-expression in the OVLT of aging male SAMP8 mice. Coronal brain sections were collected 7 days after i.c.v. injection of NGF (25 μg/kg) in 10-month-old male SAMP8 mice, and immunofluorescence staining was performed to detect GnRH-positive cells in the OVLT. **(A, B)** Representative higher-magnification fields within the OVLT (region of interest used for quantification), selected at comparable rostrocaudal levels based on OVLT anatomical landmarks. GnRH, red. Scale bars: 100 μm. **(C)** Quantification of GnRH^+^ cells within the OVLT region shown in **(A, B)** (n = 5). Data are presented as mean ± SEM and analyzed using one-way ANOVA. *P< 0.05 compared to SAMP8 mice with NGF 0 μg/kg; “ns” indicates no significant difference. **(D)** Representative double-immunofluorescence images showing GnRH (red) and Sox2 (green) co-staining in the OVLT. Scale bars: 100 μm or 20 μm.

Aging is associated with reduced hypothalamic Gnrh1 expression and a decline in GnRH neuronal function (4, 22). In rodents, GnRH-immunoreactive cells are enriched in the OVLT region. To assess whether NGF modulates GnRH-related cellular phenotypes *in vivo*, we examined the OVLT region in SAMP8 mice 7 days after a single i.c.v. injection of NGF (6.25, 12.5, or 25 μg/kg) using immunofluorescence. Our results showed an increase in the number of GnRH-positive neurons (**Figures 2A–C**). Further double-labeling for GnRH and Sox2 (a marker of neural stem/progenitor cells), revealed Sox2 expression in GnRH-positive neurons in OVLT of SAMP8 mice after NGF injection (25 μg/kg) (**Figure 2D**).

### 3.3 NGF treatment promoted htNSC differentiation toward a GnRH-associated neuronal phenotype

**Figure 3:**
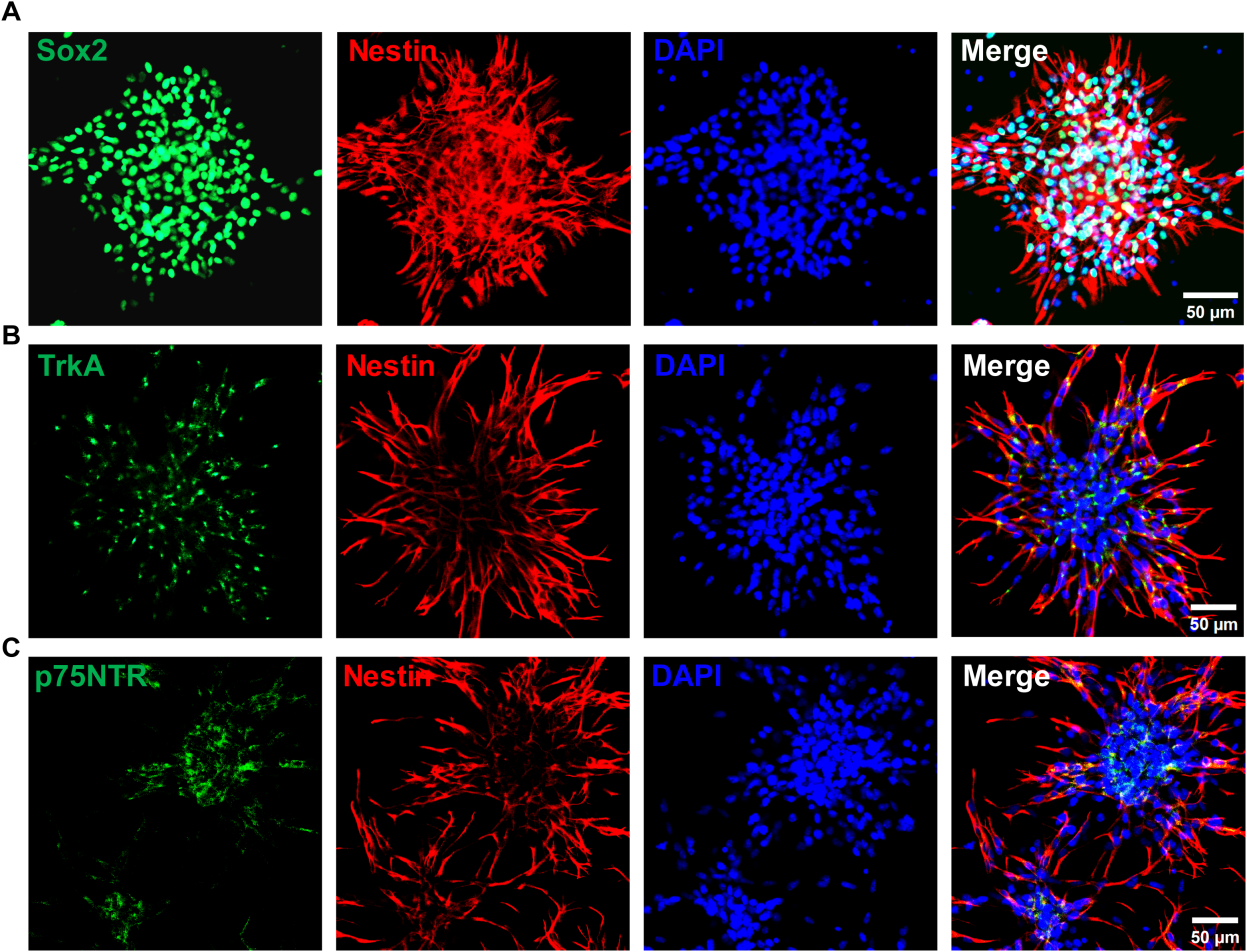
Characterization of hypothalamic neural stem cells (htNSCs) with responsiveness to neurotrophic factors. **(A)** Immunofluorescence staining showing expression of Sox2 and Nestin in primary htNSCs. **(B)** Double-labelling immunofluorescence for TrkA (green) and Nestin (red). **(C)** Double-labelling immunofluorescence for p75NTR (green) and Nestin (red). Scale bars: 50 μm.

**Figure 4:**
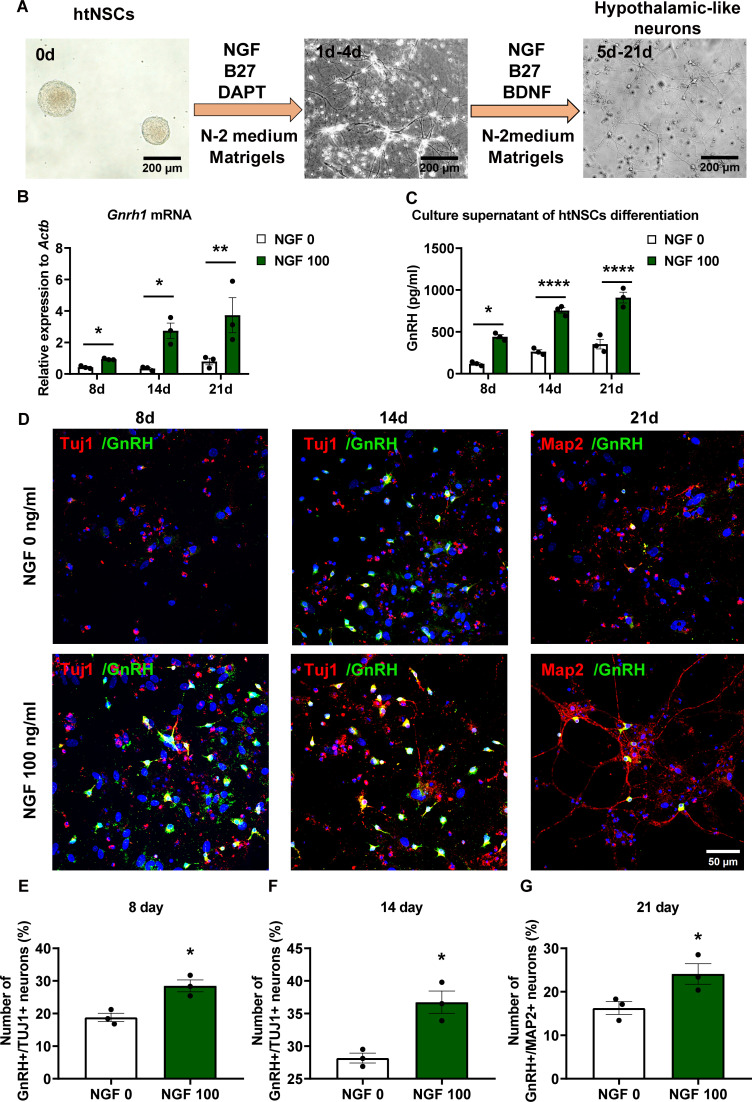
NGF promoted differentiation of 3D-cultured primary htNSCs toward a GnRH-associated neuroendocrine phenotype. **(A)** Schematic workflow illustrating the NGF-induced differentiation of primary htNSCs in 3D culture. Scale bars: 200 μm. **(B, C)** Quantification of *Gnrh1* mRNA expression and GnRH secretion in culture supernatants differentiated from 3D-cultured primary htNSCs after NGF treatment for 8, 14, and 21 days (n = 3). Two-way ANOVA. **(D)** Representative images of immunofluorescence staining for GnRH (green) and Tuj1 (red) on days 8 and 14, and GnRH (green) and Map2 (red) on day 21 in htNSCs differentiation with NGF treatment (100 ng/ml). Scale bars: 50 μm. **(E–G)** Quantification of double-labeled Tuj1^+^/GnRH^+^ (day 8 and 14) and Map2^+^/GnRH^+^ (day 21) cells relative to DAPI^+^ nuclei from five randomly selected fields per sample (n = 3). Unpaired Student’s *t*-test. Data are presented as mean ± SEM. ****P< 0.0001, **P< 0.01, *P< 0.05 compared to the control (vehicle).

**Figure 5:**
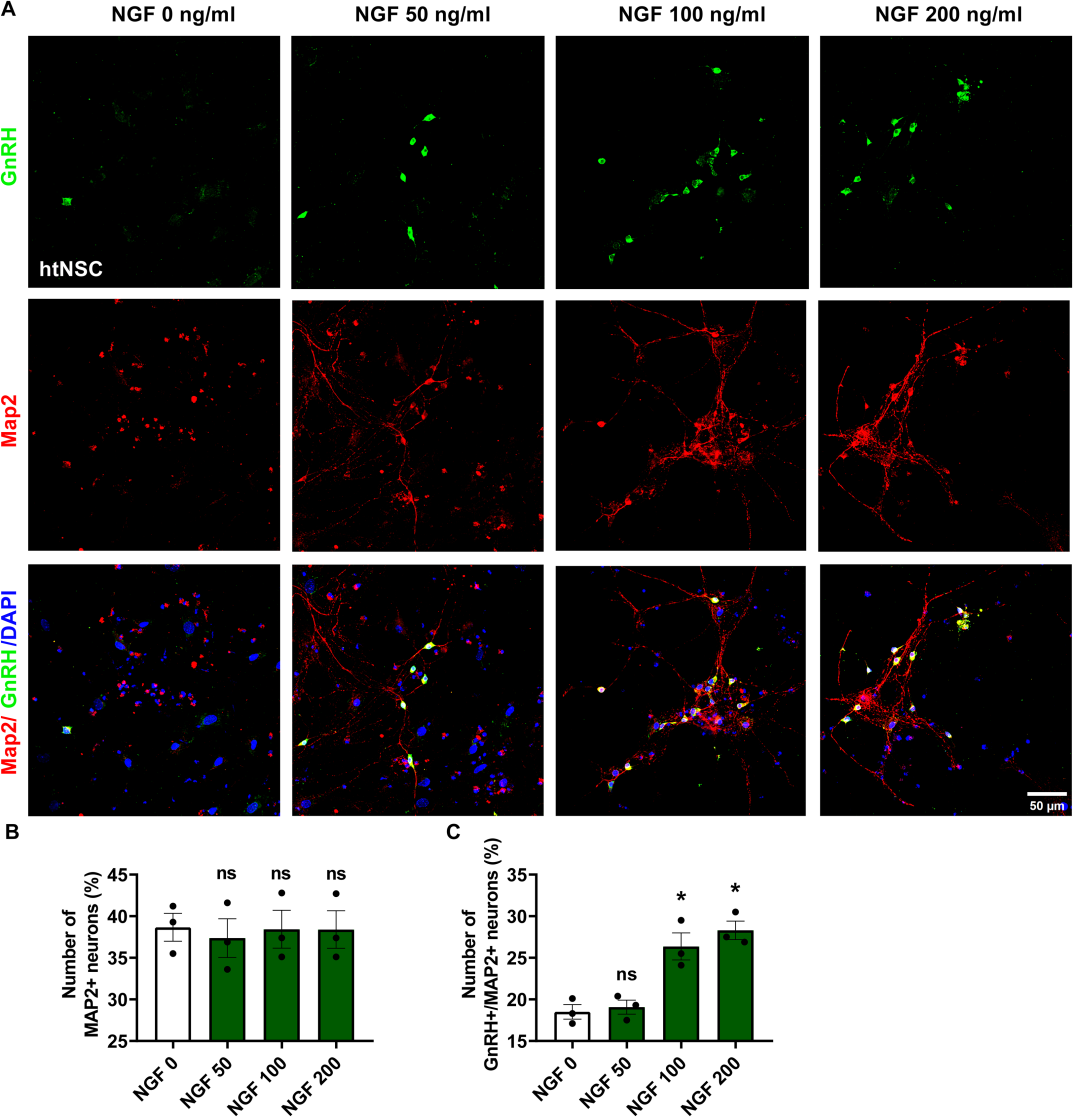
Concentration-dependent effect of NGF on GnRH-associated phenotype from 3D-htNSCs. **(A)** Immunofluorescence staining with anti-GnRH (green) and anti-Map2 (red) in differentiated htNSCs treated with different NGF concentrations (0, 50, 100, or 200 ng/ml) for 21 days. Scale bars: 50 μm. **(B, C)** Quantification of Map2^+^ and Map2^+^/GnRH^+^ cells relative to DAPI^+^ nuclei based on five randomly selected fields per sample. Data are presented as mean ± SEM and analyzed using a one-way ANOVA. *P< 0.05 compared to the control (vehicle), n = 3 independent experiments.

To investigate whether NGF drives htNSCs toward a GnRH-associated phenotype, we employed a 3D Matrigel-based culture system to induce neuronal differentiation. The isolation of primary htNSCs were confirmed by comprehensive immunocytochemical characterization. These cells exhibited robust co-expression of the canonical neural stem cell markers Sox2 and Nestin (**Figure 3A**), which validated their undifferentiated, progenitor state and established their neural stem cell (NSC) identity. Further immunostaining analysis revealed that these htNSCs expressed both the high-affinity NGF receptor TrkA and the low-affinity receptor p75NTR (**Figures 3B, C**). The comprehensive characterization of these primary htNSCs, encompassing both NSC markers and NGF receptor expression, established a reliable and physiologically relevant cellular model system for investigating the regulatory effects of NGF on htNSCs. During the two-phase induction process for htNSC differentiation (**Figure 4A**), NGF treatment (100 ng/ml) significantly upregulated *Gnrh1* mRNA expression and elevated GnRH levels in the culture supernatant on days 8, 14, and 21 (**Figures 4B, C**). Immunostaining showed co-expression of Tuj1 (a neuronal marker) and GnRH at days 8 and 14, and of Map2 (a mature neuronal marker) and GnRH at day 21 (**Figure 4D**), indicating progressive differentiation into neuron-like cells with GnRH secretory function. The control group displays immature morphology even at 21 days, with sparse and poorly branched processes. This is consistent with the limited spontaneous differentiation of htNSCs in the absence of exogenous factors. In contrast, NGF-treated cells exhibited more mature neuronal morphology, with clear dendritic structures. Quantitative analysis showed that NGF (100 ng/ml) treatment significantly enhanced htNSC differentiation into GnRH-expressing neurons, with the proportion of GnRH^+^/Tuj1^+^ cells increasing from 18.10% to 29.20% on day 8, and from 28.58% to 37.36% on day 14. Similarly, the proportion of GnRH^+^/Map2^+^ cells rose from 16.26% to 24.18% by day 21 (**Figures 4E–G**). The co-expression of GnRH and Map2 indicated that these cells formed microtubule structures and could secrete GnRH, suggesting the emergence of GnRH-associated neuronal phenotype. Further experiments with varying NGF concentrations (50 ng/ml, 100 ng/ml, 200 ng/ml) demonstrated indicates a threshold effect in the proportion of Map2^+^/GnRH^+^ neurons, while the proportion of total Map2^+^ neurons remained constant (**Figures 5A–C**). These results confirm that NGF biases htNSC differentiation toward a GnRH-associated phenotype.

### 3.4 NGF induces neuroendocrine differentiation of htNSCs via upregulation of neuropeptide signaling pathways

**Figure 6:**
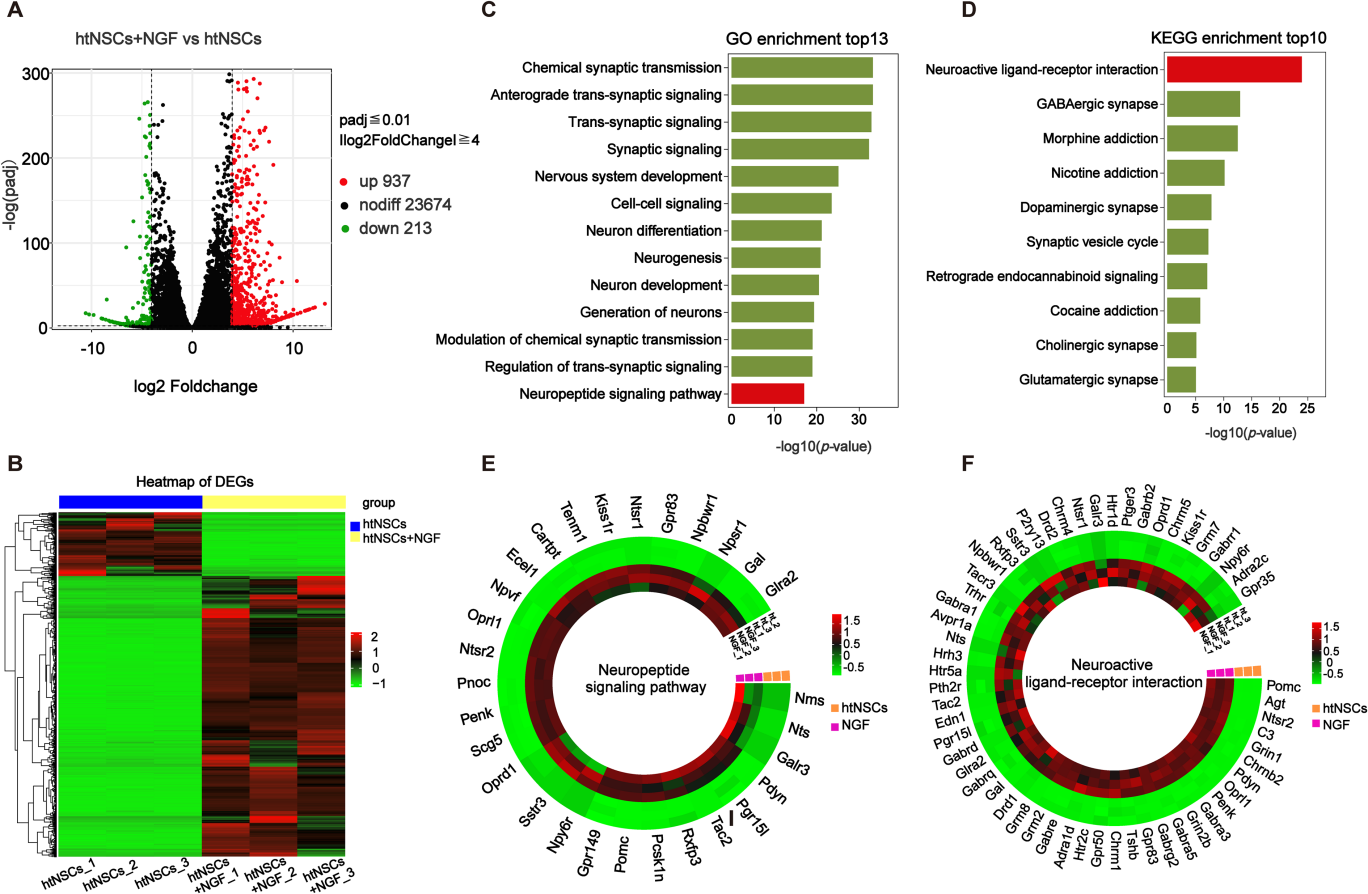
Transcriptomic profiling and functional enrichments of NGF-induced gene programs during htNSC differentiation. RNA-seq was performed on 3D-htNSCs after 21 days of NGF treatment (100 ng/ml) or vehicle control (n = 3). **(A)** Volcano plot (*p-*value vs. fold change) showing DEG between with NGF treatment and controls (vehicle) in htNSCs differentiation. Upregulated genes are highlighted in red, and downregulated genes in green. The horizontal dashed line represents padj≤ 0.01 and the vertical dashed lines represents |log2FoldChange|≥ 4. **(B)** Hierarchical clustering analysis of DEGs. **(C)** GO enrichment of the top 13 biological processes for up-regulated genes. **(D)** KEGG pathway enrichment of the top 10 upregulated terms. **(E)** Heatmap of the neuropeptide signaling pathway from GO enrichment. **(F)** Heatmap of the neuroactive ligand-receptor interaction pathways from KEGG enrichment. In both GO and KEGG enrichment, longer bars indicate more significant *p*-values. The heatmap color gradient indicates gene expression levels, with darker colors representing upregulation and lighter colors representing downregulation.

**Figure 7:**
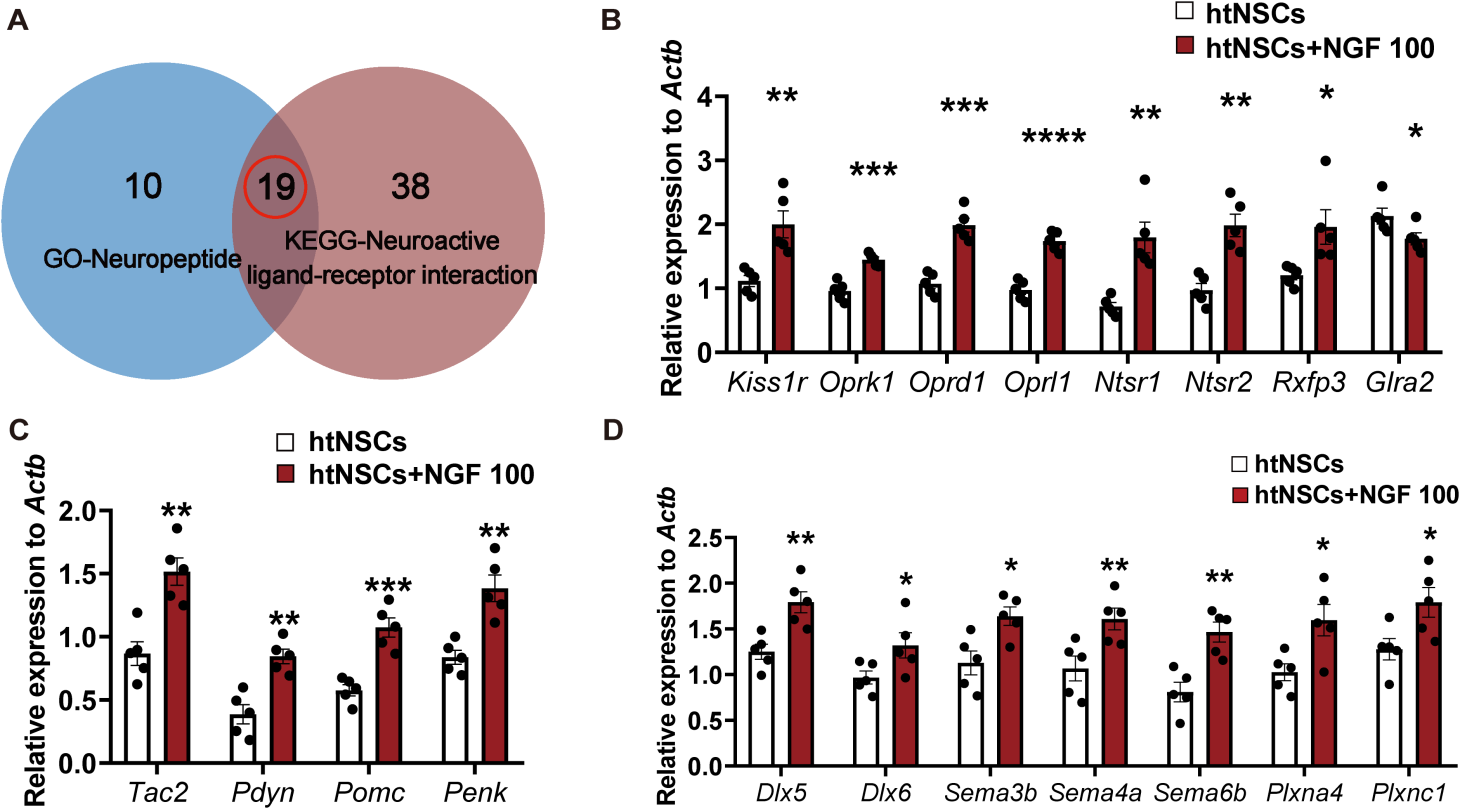
qPCR validation of neuropeptide/ligand–receptor genes and GnRH-associated phenotype markers induced by NGF in differentiating htNSCs. **(A)** Venn diagram showing 19 overlapping genes obtained from GO and KEGG enrichment gene sets. **(B, C)** qPCR validation of 4 neuropeptide and 8 receptor genes among the 19 overlaps with significant differences. **(D)** qPCR validation of representative GnRH-associated phenotype-related genes (as specified in the panel) that showed significant changes after NGF treatment. Data are presented as mean ± SEM and analyzed using unpaired Student’s *t-*test. ****P< 0.0001, ***P< 0.001, **P< 0.01, *P< 0.05 compared to control (vehicle), n = 5 independent experiments.

To elucidate the underlying molecular mechanisms, RNA sequencing was performed on htNSCs treated with NGF (100 ng/ml) for 21 days (htNSCs+NGF group) and compared to vehicle-treated controls (htNSCs group). A total of 937 upregulated genes and 213 downregulated genes were identified based on a threshold of padj≤ 0.01 and |log2FoldChange|≥ 4 in NGF-treated cells compared to controls (**Figure 6A, B**; **Supplementary Table 3**). GO enrichment analysis of the 937 upregulated genes revealed activation of pathways related to synaptic signaling, differentiation, and regeneration following NGF induction in htNSCs (**Figure 6C**; **Supplementary Table 4**). KEGG enrichment analysis further indicated that NGF treatment predominantly activated neuroactive ligand-receptor interaction signaling pathways to induce htNSC differentiation (**Figure 6D**; **Supplementary Table 5**). From the GO and KEGG enrichments, we selected genes involved in neuropeptide signaling and neuroactive ligand-receptor interactions (**Figures 6E, F**; **Supplementary Tables 6**, **S7**), yielding nineteen overlapping genes for qPCR validation (**Figure 7A**). qPCR analysis confirmed significant upregulation of four neuropeptide genes (*Tac2, Pdyn, Pomc, Penk*) and eight neuropeptide receptor genes (*Kiss1r, Oprk1, Oprd1, Oprl1, Ntsr1, Ntsr2, Rxfp3*, and *Glra2*) (**Figures 7B, C**), as well as increased expression of GnRH-associated phenotype markers (*Dlx5*, *Dlx6*, *Sema3b, Sema4a, Sema6b, Plxna4*, and *Plxnc1*) (**Figure 7D**). Genes that did not show significant changes are presented in **Supplementary Figure 1**. Notably, *Kiss1* transcripts were not detected in differentiated htNSCs by either qPCR or RNA-seq.

Together, these transcriptomic and qPCR data indicate that NGF-treated htNSCs exhibit a broad neuroendocrine-like transcriptional shift, characterized by increased expression of multiple neuropeptide and receptor genes and enrichment of neuroactive ligand-receptor interaction pathways. Consistent with our phenotypic observations, these changes support NGF-associated differentiation toward a GnRH-immunoreactive, neuroendocrine-related phenotype rather than a single lineage-specific neuronal identity.

## 4 Discussion

Aging causes testosterone decrease, associated with GnRH decline. Clinical exogenous hormone supplementation still carries potential risks, including infertility and intestinal obstruction. Stimulating GnRH-related cellular phenotypes and neuroendocrine function in the aging hypothalamus may offer an alternative strategy to improve age-related hypogonadism. Here, we report that central NGF administration enhances HPG-axis endocrine output in SAMP8 mice and is associated with increased GnRH immunoreactivity in the OVLT of aging mice and a GnRH-associated phenotype in htNSCs.

GnRH secretion is conventionally considered to be under the control of upstream kisspeptin signaling (23), and the observed increase in gonadotropin and testosterone levels in **Figure 1** could initially be interpreted as a result of kisspeptin-induced GnRH release. However, our experimental data suggest that NGF-induced elevation of sex hormones is not evidently mediated by canonical kisspeptin signaling. This conclusion is supported by our pharmacological intervention experiments, in which co-administration of kisspeptin agonists (Kp10) and antagonists (p234-p) with NGF failed to alter NGF’s ability to elevate gonadotropin levels. In contrast, our previous study (14) demonstrated that the GnRH antagonist Cetrorelix successfully abolished the NGF-induced increase in gonadotropins, indicating a direct involvement of the GnRH system. Moreover, double immunofluorescence staining confirmed the presence of the high-affinity NGF receptor TrkA on GnRH neurons (14), further supporting the possibility that NGF may act directly on these neurons. It is intriguing that LH secretion is not observed until 24 hours after NGF treatment, rather than within 8 hours. This time lag suggests the possibility of a cellular-level event such as GnRH-associated phenotype or differentiation contributing to the hormonal response. Moreover, the sustained elevation of testosterone levels over a longer period implies that NGF may not be triggering a transient, surge-like release of LH, but rather enhancing pulsatile LH secretion. To test this, subsequent experiments will use 10-min-interval tail-vein sampling over 6 h, coupled with deconvolution analysis, to quantify LH pulse frequency and amplitude before and after NGF treatment, thereby providing a definitive measure of HPG-axis functional restoration. Indeed, our current study primarily investigates whether NGF promotes the activation of GnRH-related cellular phenotypes, which could underlie the delayed hormonal response.

NGF, a classical neurotrophic factor, is well-known for its ability to support neuronal survival and regulate the proliferation and differentiation of NSCs. Changes in NGF levels in serum and brain tissues occur with aging (24, 25). Exogenous NGF administration has been reported to enhance the survival but not the proliferation of newly generated neurons in regions such as the dentate gyrus (26), the ipsilateral subependymal zone, and the injured striatum (27), all of which are established neurogenic niches in the adult brain. In recent years, the mediobasal hypothalamus has also been identified as a third neurogenic niche contributing to adult neurogenesis (28). Notably, NGF is distributed within neurogenic regions of the hypothalamus, such as the paraventricular nucleus (29, 30) and periventricular area (31), suggesting its potential involvement in hypothalamic neurogenesis, plasticity, neuroendocrine regulation, and reproductive control. Similarly, NGF’s high-affinity receptor TrkA immunoreactive cells are present in most hypothalamic areas across various species, including fish (32), mice (33), and rat (29), while the low-affinity receptor p75NTR were found in the lateral preoptic area and caudal parts of the hypothalamus (34). However, the effects of NGF infusion on hypothalamic neurogenesis remain poorly characterized. The current study provides new insights into the mechanisms through by which NGF treatment is associated with increased GnRH immunoreactivity and the emergence of a GnRH-associated phenotype in htNSC-derived cells, accompanied by enhanced HPG-axis endocrine output.

Accumulating evidence indicates that NGF is involved in the reproductive system, including the regulation of GnRH release, ovulation (35), and testis morphogenesis (36). Our previous research demonstrated that intranasal NGF administration could activate the HPG axis, ameliorating hypogonadism in male SAMP8 mice (14) and improving sperm quality in azoosperm mice (37). Given that hypothalamic GnRH serves as the primary driving force to the pituitary-gonad axis and spermatogenesis, this study focused on how NGF modulates GnRH expression and secretion. Importantly, we confirmed TrkA and p75NTR expression in primary htNSCs, supporting that the possibility that NGF-responsive differentiation of htNSCs toward a hypothalamic endocrine-like neuronal phenotype may contribute to compensatory endocrine capacity during aging.

Aging in male SAMP8 mice leads to a decline in GnRH neuronal function and testosterone levels (4, 5). Our previous study confirmed that 10-month-old SAMP8 mice exhibited significantly lower androgen levels compared to age-matched controls (14). Given that the limited permeability of the blood-brain barrier to large molecular bioproteins, peripheral NGF administration results in minimal central nervous system penetration. We directly assessed NGF’s central effects on sex hormone regulation via i.c.v. injection. This central targeting strategy is supported by NGF’s potential role in counteracting age-related hypothalamic decline. While the presence of NSCs in the adult and aging brain is well-established (38, 39), their capacity for differentiation requires specific stimuli. Critically, NGF has been demonstrated to induce NSCs differentiation into neuron-like cells *in vitro* and enhance neuronal survival. We therefore hypothesized that central injection of NGF might stimulate the differentiation of resident htNSCs into neuronal phenotype, potentially including GnRH immunoreactivity, thereby restoring neuronal function and counteracting age-related decline. To investigate this mechanism *in vitro*, we adapted established protocols for the efficient and reproducible generation of neuropeptidergic hypothalamic neurons from both human stem cells and patient-specific induced pluripotent stem cells (7, 8). Indeed, prior studies have established that FGF8 is essential for the differentiation of human pluripotent stem cells into GnRH neurons (8, 40, 41). Including an FGF8-treated group or an NGF+FGF8 combination would allow for a more comprehensive benchmarking of NGF’s effectiveness. Specifically, we employed a 3D culture system using Matrigel to enhance differentiation efficiency, mirroring advancements in directing htNSC differentiation. In the NGF-treated 3D htNSCs model, we observed significant differentiation effects. NGF exposure increased the number of GnRH-immunoreactive neuronal-like cells, indicating an enhancement of a GnRH-associated phenotype. These findings directly support our hypothesis that NGF promotes the differentiation of htNSCs toward a GnRH-associated neuronal lineage within a 3D microenvironment.

NGF treatment upregulated a broad array of genes involved in neuropeptide signaling and ligand-receptor interactions, consistent with enhanced hypothalamic neuronal differentiation programs and potential modulation of neuroendocrine function. Critically, many of these upregulated genes are known regulators of GnRH synthesis and secretion via diverse hypothalamic signaling pathways. For example, neurokinin B (coded by *Tac2*, ortholog of human *TAC3*), kisspeptin (coded by *Kiss1*), dynorphin A (coded by *Pdyn*), co-expressed by KNDy neurons, play direct roles in controlling the pulsatile GnRH release, essential for maintaining the reproductive axis (42, 43). Mutations in these genes or its receptors (e.g., *Kiss1/Kiss1r* (44, 45), *TAC3/TACR3* (46)) have been linked to impaired GnRH release and hypogonadotropic hypogonadism. Dynorphin A, through activation of the κ-opioid receptor (*Oprk1*), helps maintain reproductive hormone balance by inhibiting GnRH neuronal activity (43, 47). The *Penk* gene, encoding enkephalin, contributes to the negative regulation of the reproductive axis by suppressing GnRH neuronal activity via δ-opioid receptor signaling, particularly under metabolic stress or progesterone feedback (48, 49). Recently a study have reported that central δ-opioid receptor (*Oprd1*) and κ opioid receptor signaling pathways mediates chronic and/or acute suckling-induced LH suppression in rats during late lactation, respectively (50). However, among the opioid receptors, *Oprl1*, (primarily binds nociception) has limited known endogenous ligands, and its connection to reproductive hormone regulation remains unexplored, represent a promising avenue for future research. In addition, Semaphorin-Plexin signaling is well-established to regulate GnRH neuron development (migration, survival/maturation, and neuro-glial plasticity) (51), Dlx5/6 had been identified transcription factors essential for GnRH expression (52), we added these candidates to strengthen the mechanistic linkage between NGF-induced transcriptional remodeling and a GnRH-associated developmental program.

Beyond the KNDy system and opioids, other NGF-upregulated neuropeptides contribute to GnRH regulation. Pro-opiomelanocortin (*Pomc*) encodes peptides such as α-MSH (alpha-melanocyte-stimulating hormone) and β-endorphin, which integrate energy status and stress signals to modulate GnRH neuronal activity and reproductive function (53, 54). Neurotensin, a tridecapeptide neuropeptide, exerts its physiological effects primarily via Ntsr1 (high-affinity) and Ntsr2 (low-affinity) receptors. While early studies raised doubts about its direct role in the GnRH/LH surge (55, 56), recent evidence suggests that NTS signaling through Ntsr1 and Ntsr2 may have species-specific, context-dependent, and indirect roles in regulating the HPG axis, including GnRH regulation, ovulatory signaling, reproductive inflammation, gamete function, and hormonal homeostasis (57–61). Similarly, the relaxin−3/RXFP3 signaling pathway operates upstream of GnRH neurons and integrates stress and nutritional status signals to facilitate GnRH release, thereby activating the HPG axis (62). Conversely, while *Glra2* (glycine receptor α2 subunit) is expressed in the hypothalamus, its direct role in regulating GnRH neuronal activity or reproductive hormone secretion remains speculative and requires elucidation. Collectively, these NGF-induced gene-level alterations observed *in vitro* experiments strongly suggest that NGF orchestrates neuroendocrine control of reproductive hormones by acting directly on hypothalamic neurons and modulating key genes central to GnRH regulation.

Limitation: First, the strains of AKR/J for *in vivo* work (that is the same strain as in the 2018 publication) and BALB/c for *in vitro* work are genetically relatively distant from each other, the results can be compared, but there may be differences due to the genetic background. Second, while NGF treatment was associated with a significant increase in GnRH^+^ neurons, we did not include direct lineage tracing or proliferation assays (e.g., BrdU or Ki67 labeling) to conclusively demonstrate *de novo* neurogenesis. Accordingly, future work should incorporate these experiments to validate the neurogenic process and to distinguish neurogenesis from alternative mechanisms such as enhanced GnRH expression in pre-existing neurons. In addition, although our findings demonstrate that NGF regulates htNSCs differentiation through its receptors, the precise molecular mechanisms remain unclear. Specifically, the relative contributions of the TrkA versus p75NTR signaling pathways, as well as the interaction between NGF and other hypothalamic neuropeptides (e.g., kisspeptin, neurotensin) was not directly examined in this study and remains speculative. Future studies should clarify the molecular mechanisms by which NGF regulates htNSC differentiation across distinct physiological and pathological contexts, ideally incorporating receptor-specific blocking as well as functional readouts such as electrophysiological recordings and time-resolved secretion assays. More broadly, the roles of NGF in hypothalamic plasticity and neuroendocrine regulation, and its potential translational relevance, warrant further investigation.

## 5 Conclusion

Collectively, *in vivo* and *in vitro* data indicated that central NGF delivery is associated with enhanced GnRH immunoreactivity and promotes htNSC differentiation toward a GnRH-associated phenotype, accompanied by increased HPG-axis endocrine output in male SAMP8 mice. Direct evidence of *in vivo* neurogenesis will require BrdU/EdU/Ki67 labeling and/or lineage tracing in future studies.

## Data Availability

The datasets presented in this study can be found in online repositories. The names of the repository/repositories and accession number(s) can be found in the article/**Supplementary Material**.

## Glossary

NGF: nerve growth factor
htNSCs: hypothalamic neural stem cells
i.c.v: intracerebroventricular
LH: luteinizing hormone
FSH: follicle stimulating hormone
SAMP8: senescence accelerated mouse P8
GnRH: gonadotrophin-releasing hormone
Kp10: kisspeptin agonist kisspeptin-10
p234-p: kisspeptin antagonist p234 penetratin
OVLT: organum vasculosum of the laminae terminalis
Sox2: sex determining region Y-box 2
TrkA: tropomyosin receptor kinase A
p75NTR: p75 neurotrophin receptor
3D-htNSCs: three-dimensional hypothalamic neural stem cells
DAPT: N-[N-(3, 5-Difluorophenacetyl)-L-alanyl]-S-phenylglycine t-butyl ester
N2: N-2 Supplement
BDNF: brain-derived neurotrophic factor
Tuj1: neuron-specific class III beta-tubulin
Map2: microtubule-associated protein 2
GO: Gene Ontology
KEGG: Kyoto Encyclopedia of Genes and Genomes
*Tac2*: tachykinin 2
*Pdyn*: prodynorphin
*Pomc*: pro-opiomelanocortin-alpha
*Penk*: Preproenkephalin
*Kiss1r*: kisspeptin 1 receptor
*Oprk1*: opioid receptor kappa 1
*Oprd1*: opioid receptor delta 1
*Oprl1*: opioid receptor-like 1
*Ntsr1/2*: neurotensin receptor 1/2
*Rxfp3*: relaxin family peptide receptor 3
*Glra2*: glycine receptor alpha 2
*Npy6r*: neuropeptide Y receptor 6
*Gpr83*: G protein-coupled receptor 83
*Gal*: galanin and GMAP prepropeptide
*Pgr15l*: G protein-coupled receptor 15-like
*Nts*: neurotensin
*Npbwr1*: neuropeptides B and W receptor 1
*Sstr3*: somatostatin receptor 3
*Galr3*: galanin receptor 3
Dlx5/6: distal-less homeobox 5/6
*Sema3b/3c/3d/3e/4a/6b/6c*: semaphorin 3B/3C/3D/3E/4A/6B/6C
*Plxna4/c1*: plexin A4/C1
