# Supplementary material for: NGF administration is associated with increased GnRH immunoreactivity and a GnRH-associated phenotype in hypothalamic NSCs of aging mice
Source: Front Endocrinol (Lausanne). 2026 Apr 24;17:1736356. doi: 10.3389/fendo.2026.1736356 (PMC13153049; doi:10.3389/fendo.2026.1736356)
Supplement: Supplementary file 1 [file DataSheet1.docx]

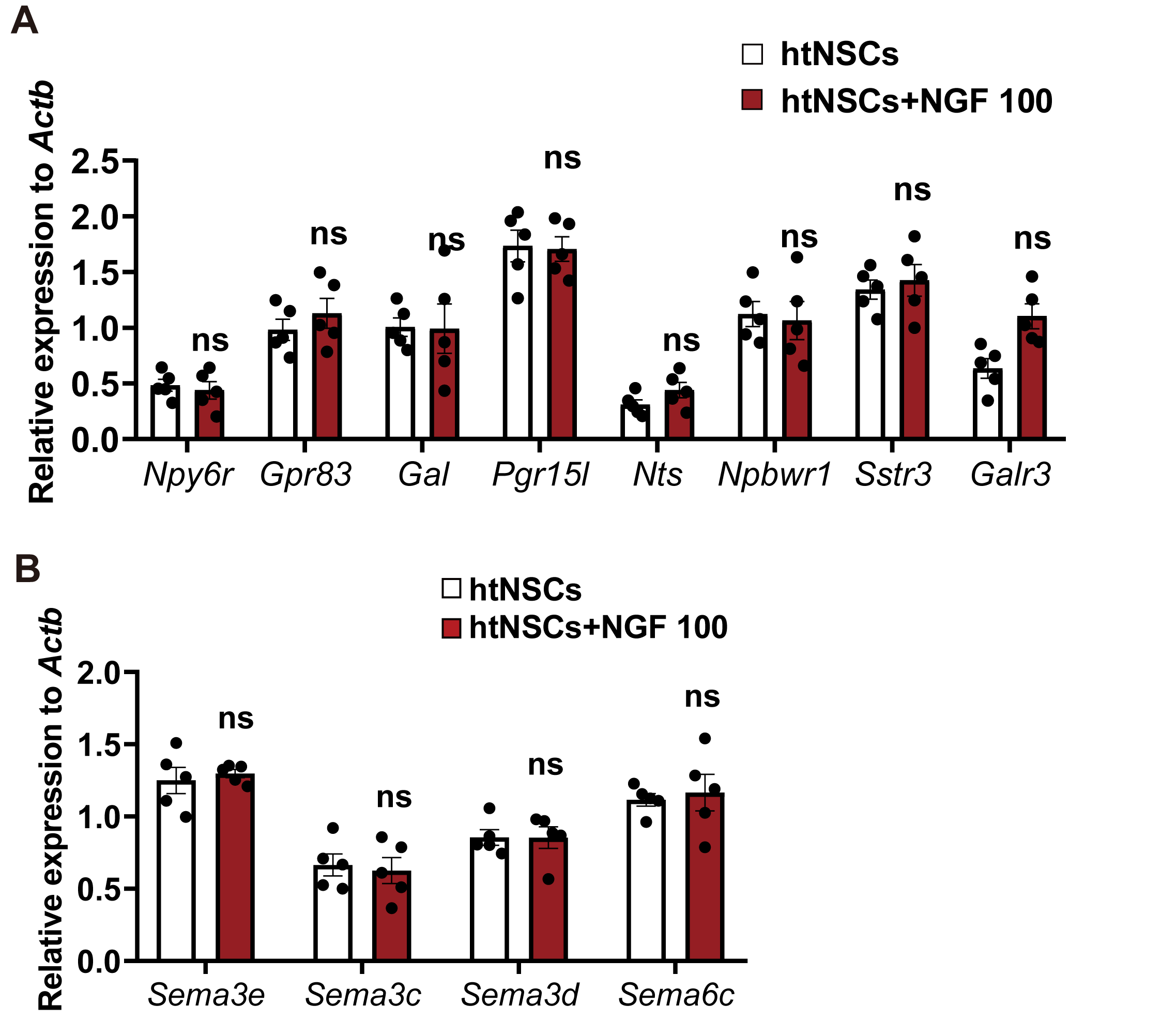


**FIGURE S1. qPCR validation of the candidates involved neuropeptide activation and GnRH-associated phenotype markers in htNSCs differentiation with NGF treatment, supplementary Figure 7. (A)** Screening of 19 genes between the Neuropeptide and Neuroactive ligand-receptor interaction pathway from GO and KEGG enrichment datasets shown in Figure 7A. 19 genes were performed qPCR analysis in 3D-htNSCs differentiation after NGF induction on day 21. Histograms of the qPCR expression of neuropeptide and receptor without significant differences. (**B**) qPCR validation of representative GnRH-associated phenotype-related genes (as specified in the panel) that showed no significant changes after NGF treatment.

Data are presented as mean ± SEM and analyzed using unpaired Student’s *t-*test. "ns" represents no statistically significant difference, for n = 5 independent experiments.
